# Genetic polymorphisms in the matrix metalloproteinase 12 gene (*MMP12*) and breast cancer risk and survival: the Shanghai Breast Cancer Study

**DOI:** 10.1186/bcr1033

**Published:** 2005-05-10

**Authors:** Aesun Shin, Qiuyin Cai, Xiao-Ou Shu, Yu-Tang Gao, Wei Zheng

**Affiliations:** 1 Department of Medicine and Vanderbilt-Ingram Cancer Center, Vanderbilt University School of Medicine, Nashville, TN, USA; 2 Department of Epidemiology, Shanghai Cancer Institute, Shanghai, China

## Abstract

**Introduction:**

Matrix metalloproteinase 12 (MMP12) is a proteolytic enzyme responsible for cleavage of plasminogen to angiotensin, which has an angiostatic effect. Using data from a population-based case–control study conducted among Chinese women in Shanghai, we evaluated the association of breast cancer risk and survival with two common polymorphisms in the *MMP12 *gene: A-82G in the promoter region and A1082G in exon, resulting in an amino acid change of asparagine to serine.

**Methods:**

Included in the study were 1,129 cases and 1,229 age-frequency-matched population controls. Breast cancer patients were followed up to determine the intervals of overall survival and disease-free survival.

**Results:**

The frequencies of the G allele in the A-82G and A1082G polymorphism among controls were 0.029 and 0.107, respectively. There were no associations between *MMP12 *polymorphisms and breast cancer risk. Patients with the AG or GG genotype of the A1082G polymorphism showed poorer overall survival (though the difference was not statistically significant) than patients with the AA genotype (hazard ratio 1.36, 95% CI 0.92 to 2.00).

**Conclusion:**

This result suggests that *MMP12 *A1082G polymorphism may be related to prognosis in breast cancer patients. Additional studies with larger sample sizes are warranted.

## Introduction

Matrix metalloproteinases (MMPs) are a family of zinc-dependent proteolytic enzymes that are involved in tumor angiogenesis, migration, and invasion as well as the regulation of immune surveillance [1,2]. With a few exceptions, the expression and activity of MMPs are increased in almost every type of human cancer and are correlated with advanced tumor stage, increased invasion and metastasis, and shortened survival [2,3]. In transplantation assays, relatively benign cancer cells acquire malignant properties when the expression of certain MMPs is up-regulated. Conversely, highly malignant cells become less aggressive when the expression or activity of certain MMPs is reduced [2].

Substrates of MMP12 are various extracellular matrix and non-extracellular-matrix proteins [4]. MMP12 may inhibit angiogenesis through cleavage of plasminogen and collagen XVIII, resulting in the generation of angiostatin and endostatin, which have an angiostatic effect [2,5,6]. On the other hand, MMP12 may promote angiogenesis by cleaving structural components of the extracellular matrix, such as collagen type IV and fibrin [2]. It has been shown that increased expression of MMP12 may reflect a favorable prognosis in a few cancers [2].

A-82G polymorphism is located on the promoter region of the *MMP12 *gene where the transcription factor activator protein 1 (AP1) binds. The A allele is associated with a higher binding affinity for AP1, resulting in higher MMP12 promoter activity *in vitro *[7]. A study showed that the A allele was associated with smaller coronary artery luminal diameter in diabetic patients treated with percutaneous transluminal coronary angiography and stent implantation [7]. In another study, however, no association was found with risk of coronary aneurysm [8]. A1082G polymorphism is located on the coding region of the hemopexin domain that is responsible for MMP12 activity. The substitution of the G allele for the A allele results in an amino acid change from asparagine (Asn) to serine (Ser) in codon 357. The functional significance of this single nucleotide polymorphism, however, has not been clearly determined. In this study, we evaluated the association of these two common polymorphisms of the *MMP12 *gene with breast cancer risk and survival in the Shanghai Breast Cancer Study.

## Materials and methods

### Study participants and design

The Shanghai Breast Cancer Study is a population-based case-control study conducted in urban Shanghai. Detailed study design and data collection procedures have been described elsewhere [9]. Briefly, cases were permanent Shanghai residents between the ages of 25 and 64 years who were newly diagnosed with breast cancer between August 1996 and March 1998. Through a rapid case ascertainment system, supplemented by the population-based Shanghai Cancer Registry, 1,602 eligible breast cancer patients were identified, and 1,459 (91.1%) completed in-person interviews using a structured questionnaire. The initial cancer diagnoses for all patients were confirmed by two senior pathologists through a review of pathological slides. Information about clinical cancer characteristics, including TNM (tumor, node, metastasis) stage, treatment for cancer, and estrogen receptor (ER) and progesterone receptor (PR) status, was obtained by medical record review using a standard protocol. The major reasons for nonparticipation were refusal (109 cases; 6.8%), death before the patient could be interviewed (17 cases; 1.1%), and our inability to locate the patient (17 cases; 1.1%)

Eligible controls were randomly selected from the Shanghai Resident Registry, which contains demographic information for all residents of urban Shanghai, and were frequency-matched on age by 5-year intervals to the predetermined age distribution of the cases reported to the Shanghai Cancer Registry from 1990 to 1993. Of the 1,734 eligible controls, 1,556 (90.3%) completed interviews. The major reasons for nonparticipation of the eligible controls were refusal (166 controls, 9.6%) or death or a prior cancer diagnosis (2 controls, 0.1%).

The structured questionnaire used for this study included information on demographic factors, menstrual and reproductive history, hormone use, previous disease history, family history of cancer, physical activity, tobacco and alcohol use, and a quantitative food-frequency questionnaire. All participants were measured for current weight, circumferences of the waist and hips, and sitting and standing height. In addition to the in-person interviews and anthropometric measurement, 10 ml blood samples were collected from 1,193 (82%) cases and 1,310 (84%) controls. These samples were processed on the same day and stored at -70°C.

The methodology for the follow-up of cancer cases was described previously [10]. All 1,459 cancer patients were followed through January 2003 with active follow-up and record linkage to the death certificates of the Vital Statistics Unit of the Shanghai Center for Disease Control and Prevention. In all, 1,290 (88.4%) patients successfully completed the follow-up interview either in person (*n *= 1,241; 85%) or by telephone (*n *= 49; 3.4%) between March 2000 and December 2002. Among them, 197 patients were deceased. Through interviews with patients – or, for deceased patients, next of kin – information on disease progress, recurrence of cancer, quality of life, and cause of death (if the patient had died) was obtained. For the remaining 169 participants, who could not be contacted in person or by phone, linkage to the death certificates was completed in June 2003. Forty deaths were identified through the linkage, and information on the date of death and cause of death was obtained. The remaining 126 subjects who had no match in the death registry were assumed to be alive on December 30, 2002, 6 months before the linkage in order to allow for a possible delay of entry of the death certificates into the registry. Four subjects had insufficient information for the record linkage and were excluded from survival analysis. Finally, 1,129 cases and 1,229 controls were included in the case-control comparison and and 1,125 cases were included in the survival analysis

### Genotyping methods

Genomic DNA was extracted from buffy coat fractions using a Puregene^® ^DNA Purification kit (Gentra Systems, Minneapolis, MN, USA) following the manufacturer's protocol. DNA concentration was measured by PicoGreen^® ^dsDNA Quantitation Kit (Molecular Probes, Eugene, OR, USA). The allelic discrimination of the *MMP12 *gene A-82G and A1082G polymorphisms were assessed with the ABI PRISM 7900 Sequence Detection Systems (Applied Biosystems, Foster City, CA, USA), using the fluorogenic 5' nuclease assay with primers and probes obtained from ABI (Assay ID: C_15880589_10 and C_785907_10). PCR was performed in a total volume of 5 μl, which contained 2.5 ng DNA, 1 × TaqMan Universal PCR Master Mix, each primer at 900 nM, and each probe at 200 nM. The thermal cycling conditions were as follows: 95°C for 10 min to activate the AmpliTaq Gold enzyme, followed by 40 cycles of 92°C for 15s and 60°C for 1 min. The fluorescence level was measured with an ABI PRISM 7900HT Sequence Detector (Applied Biosystems), resulting in clear identification of three genotypes.

The laboratory staff was blind to the identity of the subjects. Quality control samples were included in the genotyping assays. Each 384-well plate contained four water, eight CEPH 1347-02 DNA, eight blinded quality control samples, and eight unblinded quality control samples. The concordances for the blinded samples were 98% for A-82G and 100% for A1082G polymorphisms, respectively. Genotypes for polymorphisms of A-82G in the *MMP12 *gene were successfully determined for 1,118 cases and 1,223 controls and those of A1082G for 992 cases and 976 controls.

### Statistical methods

The χ^2 ^test and *t*-test were used for comparing characteristics of cases and controls. Minor genotypes AG or GG of A-82G and A1082G were combined in stratified analysis because of the small number of subjects in each category. Odds ratios and 95% confidence intervals (CIs) were derived using unconditional logistic regression models. To evaluate the association of MMP12 with survival, Cox proportional hazard models were applied after adjusting for age, TNM stages, and ER/PR status. The proportional hazard assumption of the Cox model was examined by graphic evaluation of Schoenfeld's residual plot. All *P *values presented in this paper are two-sided. SAS software was used for statistical analysis (version 9.1; SAS Institute, Cary, NC, USA).

## Results

The distribution of demographic characteristics and known breast cancer risk factors of the cases and controls are presented in Table 1. Consistent with our previous reports [9,11], reproductive risk factors such as early menarche, late menopause, and late age at the first live birth were related to increased breast cancer risk. Cases were also more likely than controls to have higher body mass index (BMI), waist-to-hip ratio, or history of breast fibroadenomas, and were less likely to have exercised regularly during the preceding 10 years. The case-control difference was not statistically significant in age and education.

The distributions of *MMP12 *A-82G and A1082G genotypes are shown in Table 2. In the controls, the genotype frequency of the A-82G polymorphism did not deviate from the Hardy–Weinberg equilibrium, but that of the A1082G genotype deviated marginally (*P *= 0.05). In the cases, the genotype frequencies of both polymorphisms deviated from the Hardy–Weinberg equilibrium; this deviation was not likely to have been due to a laboratory error, because the concordances for the quality-control samples were more than 98%. Small numbers of subjects in the GG genotypes of both polymorphisms would be a possible explanation for this deviation. The frequencies of the minor G allele of A-82G (0.029 for controls and 0.026 for cases) were substantially lower than those previously reported for Caucasian populations, which ranged from 0.11 to 0.19 [7,8,12,13], whereas the minor allele frequencies of A1082G (0.107 for controls and 0.112 for cases) were higher than in one previous report of 0.05 [12]. In agreement with an earlier report [12], we found that these two polymorphisms are not in linkage disequilibrium [14].

Overall, there were no associations of breast cancer risk with either A-82G or A1082G polymorphisms alone or in combination. The genotype association did not differ by age (<45 years vs ≥ 45 years old at the time of diagnosis), menopausal status, or family history of breast cancer (data not shown).

The frequencies of minor genotypes of both polymorphisms were not significantly higher among patients with an advanced stage of breast cancer, nor did they differ by ER/PR status (Table 3).

**Figure 1:**
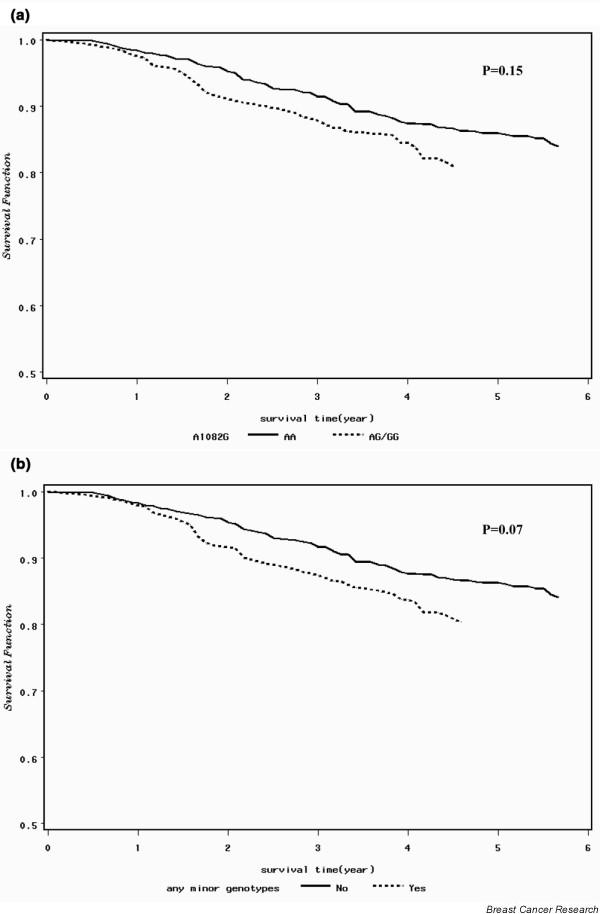
Overall survival among breast cancer patients analyzed according to A1082G polymorphism and combined A-82G and A1082G polymorphisms of the *MMP12 *gene and *P *values for survival curves derived using a log-rank test. **(a) **Patients carrying the AG or GG genotypes at the A1082G polymorphism had a lower overall survival rate than those who had the AA genotype. **(b) **Patients carrying either of the minor genotypes (AG or GG) in either A-82G or A1082G polymorphisms had a lower overall survival rate than those who had the AA genotype for both A-82G and A1082G polymorphisms.

The association of two polymorphisms of the *MMP12 *gene with breast cancer survival and disease-free survival are presented in Table 4 and Fig. 1. Patients who had the AG or GG genotypes of A1082G showed poorer overall survival than patients who had the AA genotype (hazard ratio (HR) 1.36, 95% CI 0.92 to 2.00). Compared with those who had only the AA genotypes in both A-82G and A1082G polymorphisms, patients who had one or more of the minor genotypes in these polymorphisms showed a poorer overall survival (HR 1.42, 95% CI 0.99 to 2.04). Our data did not suggest an association between *MMP12 *gene polymorphisms and disease-free survival.

## Discussion

This study suggests that two common polymorphisms (A-82G and A1082G) of the *MMP12 *gene may not be related to breast cancer risk. The A1082G polymorphism, however, may be associated with the prognosis for breast cancer patients. The association with survival seems to be independent of other clinical prognostic factors such as cancer stage or ER/PR status.

Yang and colleagues reported that overexpression of MMP12 in tumors correlated with increased survival and decreased tumor neovascularization in colorectal cancer patients [15]. Similarly, Kerkelä and colleagues reported that MMP12 expressed in macrophages in the tumor site correlated with well-differentiated cancer cells [16]. MMP12 is expressed in breast tissue and may exert its protective effect through the cleavage of plasminogen to angiostatin and of collagen XVIII to endostatin [17-19]. In addition, MMP12 is also involved in the cleavage of domain D1 of urokinase-type plasminogen activator cellular receptor, which is responsible for cell migration during tumor invasion and angiogenesis [20]. The A-82G polymorphic site is the binding site of AP1, and the A allele is related to increased MMP12 activity [3,7]. Given the functional significance of this single nucleotide polymorphism and the role of MMP12 in breast carcinogenesis, we hypothesized that this single nucleotide polymorphism may be related to breast cancer risk and survival. Our findings, however, do not support this hypothesis. The much lower frequency of the minor G allele in our study population than in Caucasian populations [7,8,12,13] substantially reduces the statistical power. Indeed, we had only 51% power to detect 30% decreased risk of AG or GG genotypes, assuming a type I error of 0.05 [21].

A1082G polymorphism of the *MMP12 *gene results in a substitution of amino acid Ser for Asn in codon 357. The function of this polymorphism has not yet been determined; however, the substitution of a hydroxylic amino acid (Ser) for an acidic amino acid (Asn) may affect the activity of the enzyme [12]. In our study, the AG or GG genotypes of A1082G polymorphism were associated with poor prognosis of breast cancer patients. This result was prominent only in overall survival, but not in disease-free survival. The information on overall survival, however, is likely to be more accurate than that on disease-free survival, because information on disease progress and recurrence was collected by interviews with patients, or kin of deceased patients, rather than by reviewing medical records. Further evaluation of this association in other populations is required.

Our study has several strengths. First, the population-based study design and the high participation rate minimize potential selection bias. Second, the homogeneous ethnicity of this population (Han Chinese) minimizes possible population stratification [22]. Third, including comprehensive lifestyle and clinical information makes it possible to consider potential confounding and interactive effects in data analysis.

## Conclusion

Our study suggests breast cancer risk may not be associated with the A-82G and A1082G polymorphisms in the *MMP12 *gene. The minor G allele in the A1082G polymorphism, however, may be related to poorer prognosis for breast cancer patients. This is the first report on the association of the *MMP12 *gene polymorphism with breast cancer risk and survival, and the results need to be confirmed in other large-scale studies.

## Abbreviations

AP = activator protein; CI = confidence interval; ER = estrogen receptor; HR = hazard ratio; MMP = matrix metalloproteinase; PR = progesterone receptor; TNM = tumor, node, metastasis.

## Competing interests

The author(s) declare that they have no competing interests.

## Authors' contributions

AS and WZ conducted data analysis and drafted the manuscript. All authors contributed to result interpretation and manuscript revision. QC performed lab assays. X-OS, Y-TG, and WZ designed the study, recruited subjects, and collected data and biological samples. WZ was the principal investigator of the study and secured the research funding. All authors read and approved the final manuscript.

## Figures and Tables

**Table 1:** Comparisons of participants with *MMP12 *genotype information.

Participant characteristics	Cases^a ^(*N *= 1,129)	Controls^a ^(*N *= 1,229)	*P*^b^
Demographic factors			
Age (years)	47.6 ± 8.0	47.2 ± 8.7	0.20
Education (% high school or more)	43.4%	43.1%	0.89
Reproductive risk factors			
Age (years) at menarche	14.5 ± 1.6	14.7 ± 1.7	<0.01
Age (years) at menopause^c^	48.2 ± 4.7	47.5 ± 4.9	0.04
Age (years) at first live birth^d^	26 ± 4.1	26.0 ± 3.8	<0.01
Other risk factors			
Breast cancer among first-degree relatives (%)	3.4	2.4	0.11
Ever had breast fibroadenoma (%)	9.7	5.1	<0.01
Body mass index (kg/m^2^)	23.5 ± 3.4	23.2 ± 3.4	0.03
Waist-to-hip ratio	0.81 ± 0.06	0.80 ± 0.06	<0.01
Physically active during past 10 years (%)	19.3	25.7	<0.01

^a^Values are presented as means ± standard deviations unless otherwise noted.

^b^Calculated from the *t*-test for continuous variables and the χ^2 ^test for categorical variables. ^c^Among postmenopausal women. ^d^Among parous women.

**Table 2:** Association of *MMP12 *A-82G and A1082G polymorphisms with breast cancer risk

MMP12 genotype	Cases	Controls	Crude OR (95% CI)	Adjusted OR^a ^(95% CI)
			
	No. (%)	No. (%)		
A-82G				
AA	1063 (95.1)	1153 (94.3)	1.0	1.0
AG	51 (4.6)	69 (5.6)	0.9 (0.6–1.2)	0.9 (0.6–1.3)
GG	4 (0.3)	1 (0.1)		
A1082G (Asn357Ser)				
AA	739 (80.2)	784 (80.3)	1.0	1.0
AG	159 (17.2)	175 (17.9)	1.0 (0.8–1.3)	1.0 (0.8–1.3)
GG	24 (2.6)	17 (1.8)		
Presence of any minor genotypes^b^				
No	687 (75.4)	731 (75.4)	1.0	1.0
Yes	224 (24.6)	239 (24.6)	1.0 (0.8–1.2)	1.0 (0.8–1.2)

^a^Adjusted for age, education, age at menarche, menopausal status, age at menopause, age at first live birth, waist-to-hip ratio, and physical activity during past 10 years. ^b^AG/GG genotypes for A-82G and AG/GG genotype for A1082G polymorphisms. CI, confidence interval; OR, odds ratio.

**Table 3:** Association of *MMP12 *polymorphisms with clinical stage and ER/PR status in breast cancer patients

MMP12 genotype				
	TNM stage No. (%)			
			
**Analyzed according to stage of cancer**	0, I, or II	III or IV		*P*
A-82G				
AA	876 (95.1)	113 (93.4)		0.42
AG/GG	45 (4.9)	8 (6.6)		
A1082G (Asn357Ser)				
AA	606 (80.5)	81 (79.4)		0.80
AG/GG	147 (19.5)	21 (20.6)		
Presence of any minor genotypes^a^				
No	563 (75.6)	74 (74.0)		0.73
Yes	182 (24.4)	26 (26.0)		
**Analyzed according to ER/PR status **	ER^+^/PR^+ ^No. (%)	ER^-^/PR^- ^No. (%)	ER^+^/PR^- ^or ER^-^/PR^+ ^No. (%)	
A-82G				
AA	401 (97.1)	188 (93.5)	144 (95.4)	0.11
AG/GG	12 (2.9)	13 (6.5)	7 (4.6)	
A1082G (Asn357Ser)				
AA	251 (77.0)	145 (84.8)	102 (83.6)	0.07
AG/GG	75 (23.0)	26 (15.2)	20 (16.4)	
Presence of any minor genotypes^a^				
No	238 (74.1)	133 (78.2)	96 (79.3)	0.41
Yes	83 (25.9)	37 (21.8)	25 (20.7)	

^a^AG/GG genotypes for A-82G and AG/GG genotype for A1082G polymorphisms. ER, estrogen receptor; PR, progesterone receptor; TNM, tumor, node, metastasis.

**Table 4:** Association of *MMP12 *A-82G and A1082G polymorphism with survival in 1,125 breast cancer patients

MMP12 genotype	Events/subjects	5-year survival rate (%)	Crude HR (and 95% CI)	Adjusted HR^a ^(and 95% CI)
**Overall survival **				
A-82G				
AA	173/1059	84.4	1.0	1.0
AG/GG	10/55	81.4	1.14 (0.60–2.16)	1.06 (0.56–2.00)
AG	9/51	82.0		
GG	1/4	75.0		
A1082G (Asn357Ser)				
AA	108/738	86.0	1.0	1.0
AG/GG	34/181	81.0	1.33 (0.90–1.95)	1.36 (0.92–2.00)
AG	32/157	79.4		
GG	2/24	91.7		
Presence of any minor genotypes^b^				
No	99/686	86.3	1.0	1.0
Yes	43/222	80.4	1.40 (0.98–2.00)	1.42 (0.99–2.04)
**Disease-free survival **				
A-82G				
AA	224/1059	78.8	1.0	1.0
AG/GG	10/55	81.7	0.87 (0.46–1.64)	0.80 (0.42–1.51)
AG	9/51	82.3		
GG	1/4	75.0		
A1082G (Asn357Ser)				
AA	151/738	79.5	1.0	1.0
AG/GG	38/181	78.8	1.04 (0.73–1.49)	1.07 (0.74–1.52)
AG	35/157	77.5		
GG	3/24	87.5		
Presence of any minor genotypes^b^				
No	142/686	79.3	1.0	1.0
Yes	47/222	78.7	1.04 (0.75–1.45)	1.06 (0.76–1.47)

^a^Adjusted for age, TNM stage, and ER/PR status. ^b^AG/GG genotypes for A-82G and AG/GG genotype for A1082G polymorphisms. CI, confidence interval; ER, estrogen receptor; HR = hazard ratio; PR, progesterone receptor; TNM, tumor, node, metastasis.

## References

[B1] Vu TH, Werb Z (2000). Matrix metalloproteinases: effectors of development and normal physiology. Genes Dev.

[B2] Egeblad M, Werb Z (2002). New functions for the matrix metalloproteinases in cancer progression. Nat Rev Cancer.

[B3] Ye S (2000). Polymorphism in matrix metalloproteinase gene promoters: implication in regulation of gene expression and susceptibility of various diseases. Matrix Biol.

[B4] Chakraborti S, Mandal M, Das S, Mandal A, Chakraborti T (2003). Regulation of matrix metalloproteinases: an overview. Mol Cell Biochem.

[B5] Dong Z, Kumar R, Yang X, Fidler IJ (1997). Macrophage-derived metalloelastase is responsible for the generation of angiostatin in Lewis lung carcinoma. Cell.

[B6] Cornelius LA, Nehring LC, Harding E, Bolanowski M, Welgus HG, Kobayashi DK, Pierce RA, Shapiro SD (1998). Matrix metalloproteinases generate angiostatin: effects on neovascularization. J Immunol.

[B7] Jormsjo S, Ye S, Moritz J, Walter DH, Dimmeler S, Zeiher AM, Henney A, Hamsten A, Eriksson P (2000). Allele-specific regulation of matrix metalloproteinase-12 gene activity is associated with coronary artery luminal dimensions in diabetic patients with manifest coronary artery disease. Circ Res.

[B8] Lamblin N, Bauters C, Hermant X, Lablanche JM, Helbecque N, Amouyel P (2002). Polymorphisms in the promoter regions of MMP-2, MMP-3, MMP-9 and MMP-12 genes as determinants of aneurysmal coronary artery disease. J Am Coll Cardiol.

[B9] Gao YT, Shu XO, Dai Q, Potter JD, Brinton LA, Wen W, Sellers TA, Kushi LH, Ruan Z, Bostick RM (2000). Association of menstrual and reproductive factors with breast cancer risk: results from the Shanghai Breast Cancer Study. Int J Cancer.

[B10] Shu XO, Gao YT, Cai Q, Pierce L, Cai H, Ruan ZX, Yang G, Jin F, Zheng W (2004). Genetic polymorphisms in the TGF-beta 1 gene and breast cancer survival: a report from the Shanghai Breast Cancer Study. Cancer Res.

[B11] Shu XO, Cai Q, Gao YT, Wen W, Jin F, Zheng W (2003). A population-based case-control study of the Arg399Gln polymorphism in DNA repair gene XRCC1 and risk of breast cancer. Cancer Epidemiol Biomarkers Prev.

[B12] Joos L, He JQ, Shepherdson MB, Connett JE, Anthonisen NR, Pare PD, Sandford AJ (2002). The role of matrix metalloproteinase polymorphisms in the rate of decline in lung function. Hum Mol Genet.

[B13] Zhang B, Dhillon S, Geary I, Howell WM, Iannotti F, Day IN, Ye S (2001). Polymorphisms in matrix metalloproteinase-1, -3, -9, and -12 genes in relation to subarachnoid hemorrhage. Stroke.

[B14] Devlin B, Risch N (1995). A comparison of linkage disequilibrium measures for fine-scale mapping. Genomics.

[B15] Yang W, Arii S, Gorrin-Rivas MJ, Mori A, Onodera H, Imamura M (2001). Human macrophage metalloelastase gene expression in colorectal carcinoma and its clinicopathologic significance. Cancer.

[B16] Kerkelä E, Ala-aho R, Klemi P, Grenman S, Shapiro SD, Kahari VM, Saarialho-Kere U (2002). Metalloelastase (MMP-12) expression by tumour cells in squamous cell carcinoma of the vulva correlates with invasiveness, while that by macrophages predicts better outcome. J Pathol.

[B17] Heppner KJ, Matrisian LM, Jensen RA, Rodgers WH (1996). Expression of most matrix metalloproteinase family members in breast cancer represents a tumor-induced host response. Am J Pathol.

[B18] Zucker S, Vacirca J (2004). Role of matrix metalloproteinases (MMPs) in colorectal cancer. Cancer Metastasis Rev.

[B19] Ferreras M, Felbor U, Lenhard T, Olsen BR, Delaissé JM (2000). Generation and degradation of human endostatin proteins by various proteinases. FEBS Lett.

[B20] Koolwijk P, Sidenius N, Peters E, Sier CF, Hanemaaijer R, Blasi F, van Hinsbergh VW (2001). Proteolysis of the urokinase-type plasminogen activator receptor by metalloproteinase-12: implication for angiogenesis in fibrin matrices. Blood.

[B21] Schlesselman JJ (1982). Sample size. Case-Control Studies.

[B22] Freedman ML, Reich D, Penney KL, McDonald GJ, Mignault AA, Patterson N, Gabriel SB, Topol EJ, Smoller JW, Pato CN (2004). Assessing the impact of population stratification on genetic association studies. Nat Genet.

